# Time-limited involvement of caudal anterior cingulate cortex in trace eyeblink conditioning retrieval is dependent on conditioned stimulus intensity

**DOI:** 10.1371/journal.pone.0191320

**Published:** 2018-01-25

**Authors:** Xuan Li, Guang-yan Wu, Juan Yao, Yi Yang, Jian-ning Ye, Jian-feng Sui

**Affiliations:** 1 Department of Physiology, College of Basic Medical Science, Third Military Medical University, Chongqing, P.R. China; 2 Experimental Center of Basic Medicine, College of Basic Medical Science, Third Military Medical University, Chongqing, P.R. China; 3 Department of Neurology, Xinqiao Hospital, Third Military Medical University, Shapingba District, Chongqing, P.R. China; Tokai University, JAPAN

## Abstract

The medial prefrontal cortex (mPFC) has been widely investigated for its roles in learning and memory. The present study investigated the time-limited involvement of the caudal anterior cingulate cortex (cACC) of the mPFC in the retrieval process for a simple associative motor learning, trace eyeblink conditioning (tEBC), using a 75 dB or 100 dB tone as the conditioned stimulus (CS). The GABA_A_ receptor agonist muscimol was injected into the cACC of guinea pigs at 1 day or 4 weeks after tEBC acquisition. When muscimol was administered 1 day after tEBC acquisition, the conditioned response (CR) of the 75 dB group was severely impaired, whereas the CR of the 100 dB group exhibited no significant change relative to the control. When muscimol was administered 4 weeks after tEBC acquisition, the CR was impaired in both the 75 dB and 100 dB groups. This study indicate that the cACC of the mPFC is necessary for recent retrieval of tEBC with a low-intensity CS but not of tEBC with a high-intensity CS, whereas for remote retrieval of tEBC, the cACC of the mPFC is essential regardless of whether the CS intensity is high or low. These results support a conditional role for the mPFC in modulating recent retrieval of tEBC and a persistent role for its involvement in remote retrieval of tEBC.

## Introduction

Eyeblink conditioning (EBC) is an excellent model system to explore the neural substrates underlying associative motor learning and memory. This model typically involves paired presentation of a conditioned stimulus (CS) and an unconditioned stimulus (US) [1–5]. Two variants of EBC, the trace and delay paradigms, differ in the temporal relationship between the CS and US. In delay eyeblink conditioning (dEBC), the CS precedes and co-terminates with the US; in trace eyeblink conditioning (tEBC), the two stimuli are separated by a stimulus-free trace interval. While dEBC can be well established using cerebellar-brainstem circuits, tEBC also requires several forebrain structures, such as the prefrontal cortex (PFC) and the hippocampus [6–8].

The PFC has long been known to play a crucial role in many behavioral and cognitive functions, and the medial PFC (mPFC) has also been found to be involved in associative motor learning, such as EBC [9, 10]. Recent studies have demonstrated that mPFC neurons exhibit sustained firing during the trace interval of tEBC, which might facilitate formation of CS-US trace associations by supporting working memory processes [6, 11]. Although it is widely accepted that the mPFC may be necessary for the acquisition and retrieval of tEBC, recent studies demonstrate that various factors such as US intensity and CS duration may affect the involvement of the mPFC in modulation of tEBC [12–14]. For example, Oswald et al. reported that lesions of the mPFC impair tEBC specifically when US intensity is low but not when US intensity is high [13], suggesting that a US with high intensity may recruit additional brain areas (e.g., the amygdala) to facilitate the functional association between the mPFC and subcortical structures (e.g., the cerebellum) during the trace period; McLaughlin et al. revealed that mPFC lesions impair tEBC when the CS duration is 500 ms but not when it is 100 ms [10]. In addition, our previous studies demonstrated that mPFC and its projections to the pontine nuclei are essential for dEBC when the CS intensity is low but not when the CS intensity is high [15–17]. Moreover, the mPFC has been found to play a time-limited role in memory. These findings, taken as a whole, lead us to hypothesize that the involvement of the mPFC in tEBC modulation may be both time-limited and CS intensity dependent.

The mPFC consists of several highly interconnected regions, including the anterior cingulate cortex (ACC), prelimbic cortex (PL) and infralimbic cortex (IL). The caudal ACC (cACC) were has been reported to be critically involved in associative learning tasks [18–21]. However, whether the cACC is conditionally or persistently required for modulation of recent and remote retrieval of tEBC remains unclear.

Here, we test our hypothesis by investigating the time-limited involvement of the cACC of the mPFC in tEBC retrieval with a 75 dB or 100 dB tone CS by reversibly inactivating the cACC with muscimol infusion at 1 day or 4 weeks after tEBC acquisition. The results show that the cACC of the mPFC is necessary for recent retrieval of tEBC with a low-intensity CS but not of tEBC with a high-intensity CS and that the cACC is essential for remote retrieval of tEBC with a low-intensity CS and with a high-intensity CS. Our results support a conditional role for the mPFC in modulating recent retrieval of tEBC and a persistent role for its involvement in remote retrieval of tEBC.

## Materials and methods

### Subjects

The subjects were 16 adult male albino Dunkin-Hartley guinea pigs, weighing 450–550 g (3–4 months old). The guinea pigs were housed in standard plastic cages individually, with free access to food and water. A 12:12 light/dark cycle was used, and all experiments were performed during the daylight portion of the light/dark cycle. The procedures were approved by the Animal Care Committee of the Third Military Medical University and were performed in accordance with the principles outlined in the National Institutes of Health Guide for the Care and Use of Laboratory Animals. All possible effort was made to optimize the comfort and to minimize the use of the animals.

### Surgery

All surgery was performed under aseptic conditions. The animals were general anaesthetized with a mixture of ketamine (80 mg/kg, i.p.) and xylazine (5 mg/kg, i.p.), and then their heads were positioned in a stereotaxic apparatus. According to an atlas of the guinea pig brain [21], the skull above the prefrontal cortex was removed with a dental drill. A pair of stainless steel guiding cannulaes (external diameter: 0.6 mm, internal diameter: 0.4 mm) were stereotaxically implanted into the bilateral caudal anterior cingulate cortex of mPFC, and their tips were directed to the following coordinates: anteroposterior (AP) +2.0 mm to bregma, mediolateral (ML) 1.0 mm to midline suture, dorsoventral (DV) −2.5 mm to the skull surface. A stainless steel stylet (diameter: 0.3 mm) was inserted into the guiding cannula to prevent occlusion. A plastic headstage was placed behind the guiding cannula to secure the animal’s head. The guiding cannula and the plastic headstage were fixed on the skull with dental cement. A nylonloop was sutured into animal’s left upper eyelid, its tail linked to a movement-measuring device to measure the eyeblink responses. The animals were allowed one week to recover from the surgery before behavioral training.

### Apparatus and procedures

Experiments were performed in a sound- and light-attenuated chamber. During the experiment, animal was restrained in a plexiglas retainer. A high-resolution spring return potentiometer (JZ101, XH, Beijing, China) was linked to the nylonloop which was sutured into the animal’s left upper eyelid to measure the eyeblink responses. A speaker was placed 60 cm above the animal for delivering the tone CS. A plastic pipe was placed 1.0 cm from the animal’s left eye for delivering a corneal air-puff US. The signal of eyeblink responses were recorded and sampled at 1000 Hz by a data acquisition system (RM6280C, Cheng Yi, Chengdu, China).

The animals were divided into 2 groups: the 75 dB group (n = 8) and the 100 dB group (n = 8). Before behavioral training, the animals received two habituation sessions for adapting to the experimental environment. Following habituation sessions, animals of the 75 dB and 100 dB groups were sequentially trained for 10 daily sessions. Daily sessions consisted of 10 blocks of nine CS-US paired trials and one CS-alone trial, for a total of 100 trials per session. The CS was a 100-ms duration, 3-kHz pure tone, with an intensity of either 75 dB or 100 dB. The US was a 100-ms duration, 3.0-psi corneal airpuff. In CS-US paired trials, CS was followed by a 350-ms trace interval, before the onset of US. The trials were separated by a variable interval between 20 and 40 s that averaged 30 s. Presentations of the CS and US were controlled by a custom computer-based system.

One day and 4 weeks after daily training, the animals of two groups underwent 2 consecutive daily sessions of muscimol and artificial cerebrospinal fluid (aCSF) infusion (muscimol in the first session, aCSF in the second session). In recent post-training infusion, 3 blocks of training were performed prior to muscimol or aCSF infusion to establish the eyeblik baseline, the subsequent 7 blocks of training began at 20 min after muscimol or aCSF infusion. In remote post-training infusion, 2 blocks of training were performed prior and post to muscimol and aCSF infusion respectively.

### Drug and microinfusions

Animals were placed in plexiglas retainer with head restraining. The stylet was removed from the guiding cannula and replaced by a stainless steel infusion cannula (external diameter: 0.3 mm, internal diameter: 0.2 mm), which was connected to polyethylene tubing. The tip of the infusion cannula extended 0.5 mm beyond the tip of the guiding cannula, and the infusion position was directed to the following coordinates: AP +2.0 mm, ML 1.0 mm, DV −3.0 mm. The animals were bilaterally infused with 1.0 μL of Muscimol (Sigma–Aldrich, St. Louis, MO, USA; 1 mM, pH 7.4) or aCSF at a speed of 0.5 μL/ min via a microsyringe, which was connected to polyethylene tubing. After infusion, the infusion cannula was kept in place for 5 min and then it was replaced by the style.

### Histology

At the end of the experiment, the animals were deeply anesthetized with sodium pentobarbital (80 mg/kg, i.p.) and transcardially perfused with 0.9% saline followed by 4% paraformaldehyde. After perfusion, the brains were stored in paraformaldehyde solution for several days and infiltration with 30% sucrose / 4% paraformaldehyde solution. The brains were then frozen and coronally sectioned at 30 μm, and stained with cresyl violet. The sections were subsequently checked using a light microscope (SMZ1500, Nikon, Tokyo, Japan), and their images were acquired using a digital camera (DXM1200F, Nikon, Tokyo, Japan). The locations of the infusion cannula tips were drawn onto plates from the stereotaxic atlas of the guinea pig brain.

### Behavioral data and statistical analysis

**Fig 1:**
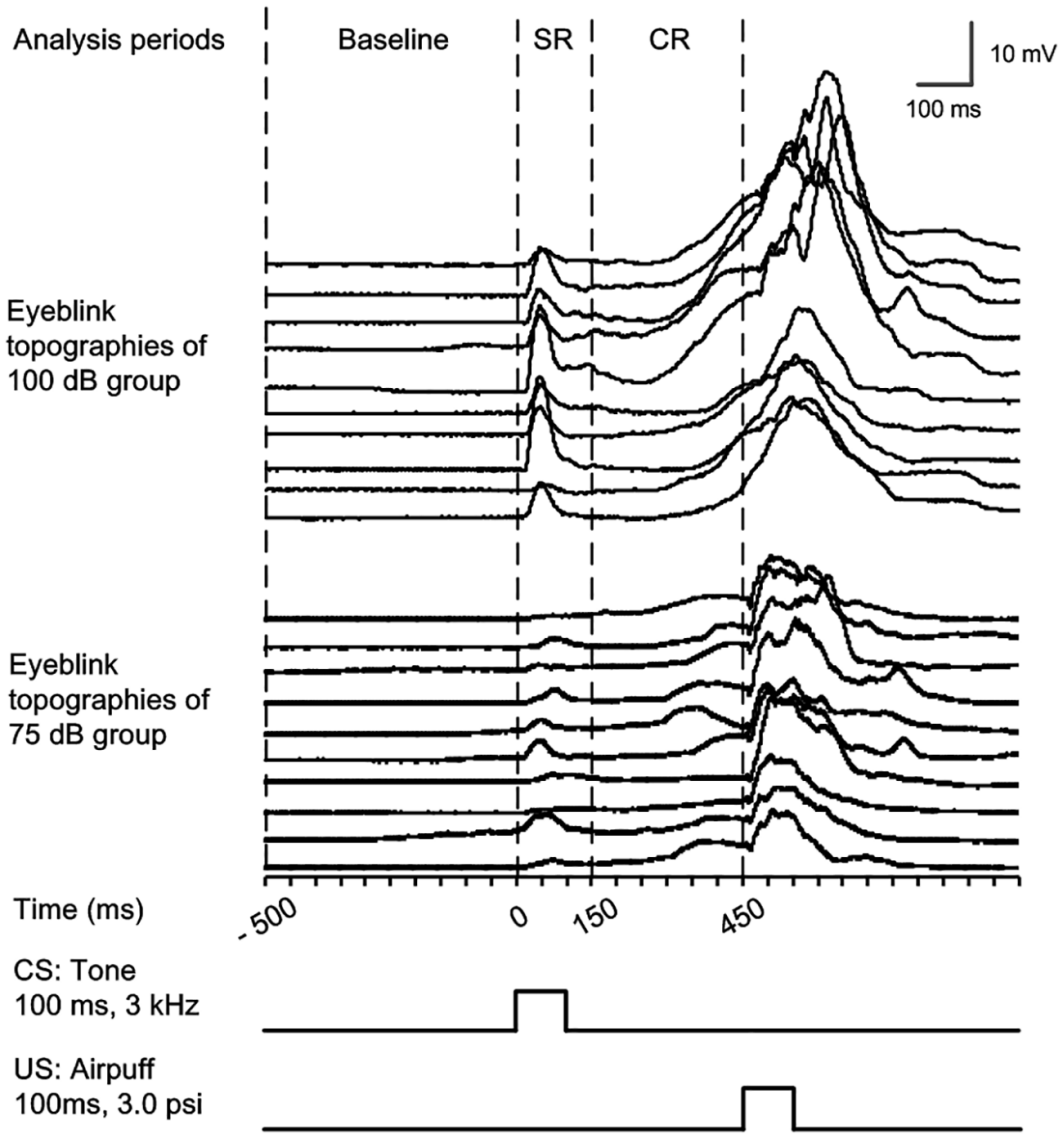
The temporal relationships of the analysis periods during trace eyeblink conditioning. The eyeblink waterfall plots of 75 dB and 100 dB groups contains ten trials across ten blocks during last acquisition training session of a representative guinea pig of the two groups respectively. In each trial, we analyzed the parameters of the startle eyeblink response (SR; analysis period within 0–150 ms after the CS onset) and conditioned eyeblink response (CR; analysis period within 150–450 ms after the CS onset). These responses were based on the average amplitude of the baseline within 0–500 ms period prior to the onset of the CS.

Each CS–US paired or pseudo-paired trial presented during recording was subdivided into three discontinuous analysis periods (Fig 1): (1) the “baseline” period, 0–500 ms before the CS onset; (2) the “eyeblink startle response (SR)” period, 0–150 ms after the CS onset; (3) the “CR” period, 150–450 ms after the CS onset

The eyeblink activity in SR and CR periods were compared to the mean of amplitude in baseline period. A significant eyelid movement was defined as greater than the baseline amplitude 5 times standard deviation of the baseline activity, and lasted ≥15 ms. Any significant eyelid movement during the corresponding periods was counted as a SR or a CR. The percentage of SR and CR was defined as the ratio of the number of trials containing the SR and CR to the total number of valid trials. Once a CR was detected, the CR peak amplitude was recorded as the maximum amplitude change from baseline during the CR period and the CR peak latency was recorded as the time interval from the CS onset until the peak of the CR. The mean CR peak amplitude is a mean value of the CR peak amplitude for the trials with a CR and the mean CR peak latency is a mean value of the CR peak latency for the trials with a CR.

All data were expressed as the mean ± SEM. Statistical significance was determined by a two-way ANOVA with repeated measures followed by Tukey post-hoc test, or by a Paired-Sample T test using SPSS statistical software. Differences with a p value of < 0.05 were considered significant.

## Result

### Infusion cannula tips placements

**Fig 2:**
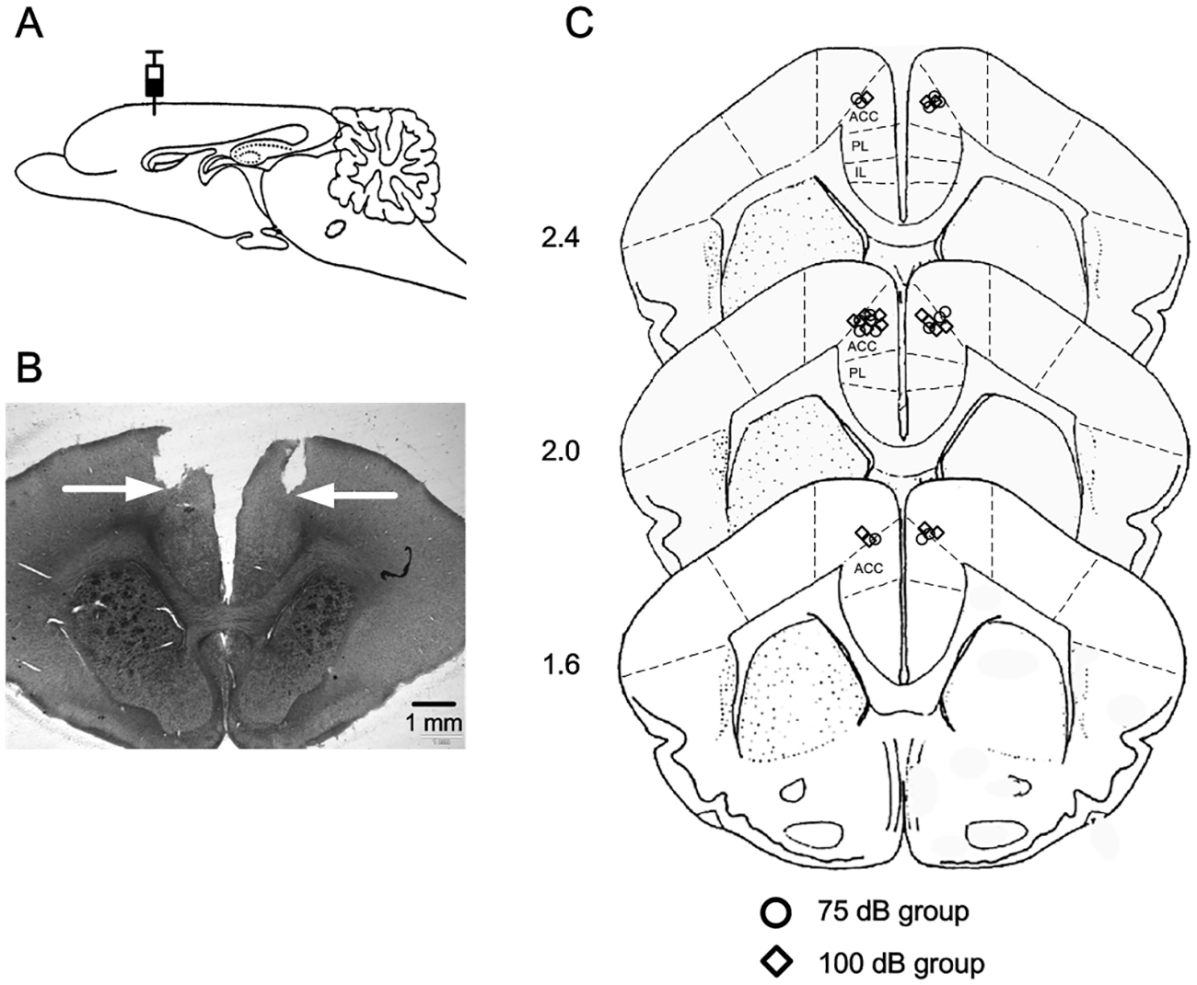
Histological reconstruction of the infusion cannula tips. (A) Diagram of the sagittal section of guinea pig brain, showing the infusion sites. (B) A representative photomicrograph of coronal sections showing the placement of guiding cannula tips in the mPFC, arrows indicate the locations of the infusion cannula tips. (C) Schematic illustration of estimated infusion locations based on the tips location of infusion cannula in the 75 dB (n = 8, blank circle) and 100 dB (n = 8, blank square) groups. Numbers to the left represent distance (mm) from the coronal sections to the bregma. (ACC: anterior cingulate cortex; PL: prelimbic cortex; IL: infralimbic cortex).

Placements of infusion cannula tips were checked before the behavioral analysis, and all of the infusion cannula tips placements of the 75 dB group (n = 8, blank circle) and 100 dB group (n = 8, blank square) groups were in the mPFC, and centered in caudal anterior cingulate cortex. Fig 2A shows a representative brain section from a guinea pig used in the present experiment. Fig 2B diagrams the cannula tip placements of the animals in the two groups.

### Acquisition of trace eyeblink conditioning

**Fig 3:**
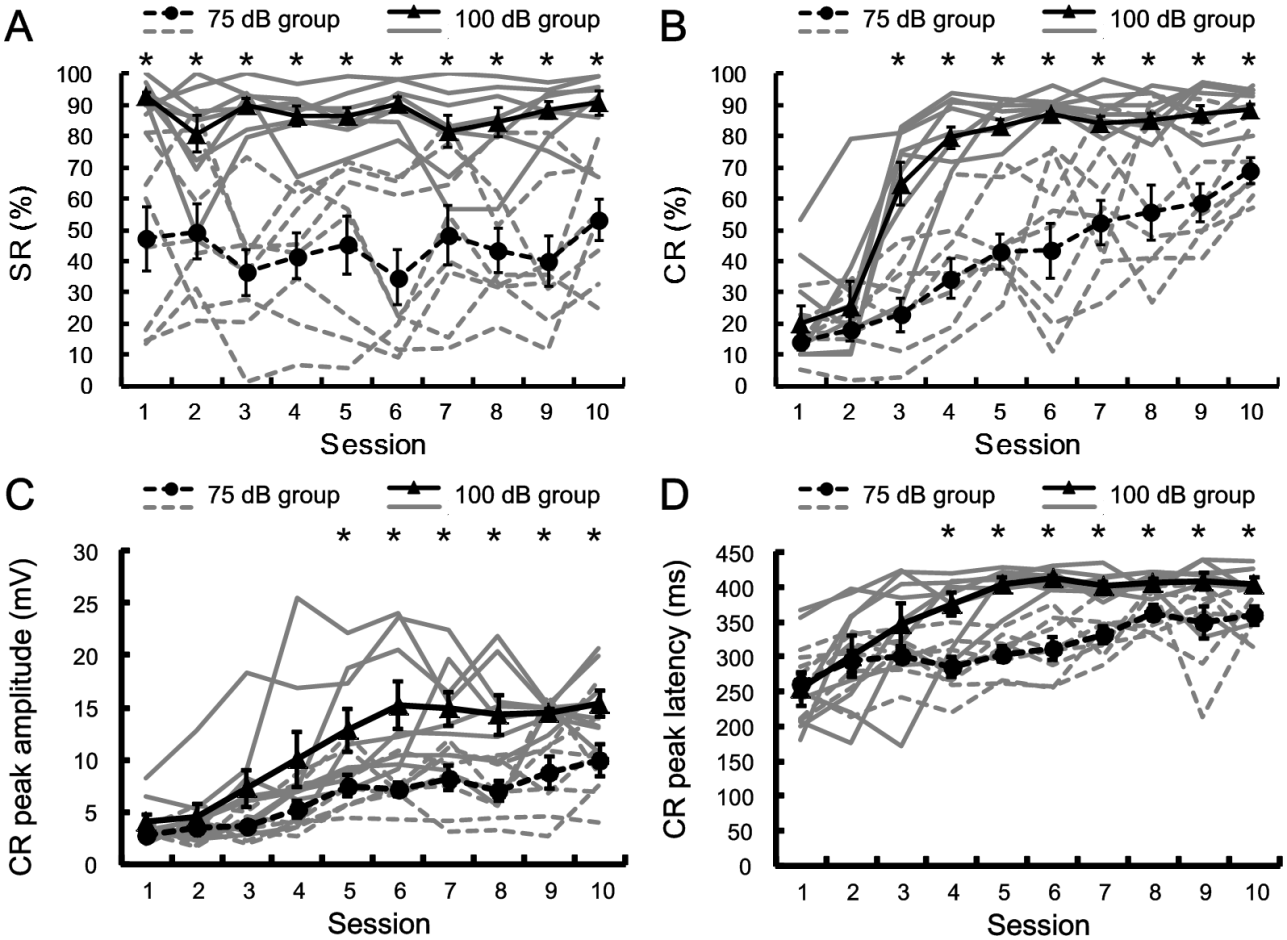
Learning curves of trace eyeblink condition with 100 dB tone CS and 75 dB tone CS. The individual and mean value ± SEM for the percentage of SR (A), the percentage of CR (B), CR peak amplitude (C), and CR peak latency (D) in the 75 dB group (n = 8; individual: dash gray line; mean value: black solid circle) and 100 dB group (n = 8; individual: solid gray line; mean value: black solid triangle). Error bars represent the SEM. Two-way repeated measures ANOVA revealed that there were significant effects of the group on the SR%, CR%, CR peak amplitude and CR peak latency [SR%: F(1, 14) = 36.902, p < 0.001; CR%: F(1, 14) = 58.842, p < 0.001; CR peak amplitude: F(1, 14) = 12.009, p = 0.004; CR peak latency: F(1, 14) = 27.71, p < 0.001]. Significant effects of the session were found in CR%, CR peak amplitude and CR peak latency [CR%: F(9, 126) = 40.155, p < 0.001; CR peak amplitude: F(9, 126) = 23.408, p < 0.001; CR peak latency: F(9, 126) = 15.140, p < 0.001]. Differences between groups were statistically significant in the indicated sessions. *p < 0.05; two-way repeated measure ANOVA followed by post-hoc test.

Fig 3 shows behavioral data of acquisition of the tEBC of the 75 dB and 100 dB groups. To investigate the startle responses of the animals, we analyzed the SR% across the 10 sessions of the two groups. As expected, compared with that in 75 dB group, animals in 100 dB group show more SR (Fig 3A). This was confirmed by two-way repeated measure ANOVA on the SR%, there was a significant effect of group [F(1, 14) = 36.902, p < 0.001], but no significant effect of session [F(9, 126) = 0.963, p = 0.474] and group by session interaction [F(9, 126) = 1.736, p = 0.087]. As shown in Fig 3B, the average CR% increased as a function of sessions for both the 75 dB and the 100 dB groups, CR learning curves for individual animals show that all animals in both 75 dB and 100 dB groups exhibit fast or slow acquisition of CRs, however, the learning curves in 75 dB group has a more modest slope. A two-way repeated measures ANOVA on the CR% revealed that there were significant effects of the group [F(1, 14) = 58.842, p < 0.001], the session [F(9, 126) = 40.155, p < 0.001] and group by session interaction [F(9, 126) = 4.528, p < 0.001]. Furthermore, post-hoc test revealed that the CR% of the 100 dB group was significantly higher than that of the 75 dB group on sessions 3–10.

CR peak amplitude and CR peak latency of the 75 dB and 100 dB groups were analyzed to investigate the effect of CS intensity on the CR pattern. The CR peak amplitude and CR peak latency across the 10 sessions are illustrated in Fig 3C and 3D, respectively. Two-way repeated measures ANOVA revealed the significant effects of the group [CR peak amplitude: F(1, 14) = 12.009, p = 0.004; CR peak latency: F(1, 14) = 27.71, p < 0.001], the session [CR peak amplitude: F(9, 126) = 23.408, p < 0.001; CR peak latency: F(9, 126) = 15.140, p < 0.001] and group by session interaction [CR peak amplitude: F(9, 126) = 2.739, p = 0.006; CR peak latency: F(9, 126) = 2.992, p = 0.003] on the CR peak amplitude and on the CR peak latency. Post-hoc test revealed that the CR peak amplitude of the 100 dB group was significantly greater than that of the 75 dB group on sessions 5–10, and the CR peak latency of the 100 dB group was significantly greater than that of the 75 dB group on sessions 4–10.

### Effects of cACC inactivation on the recent and remote retrieval of tEBC with 75 dB and 100 dB tone CS

**Fig 4:**
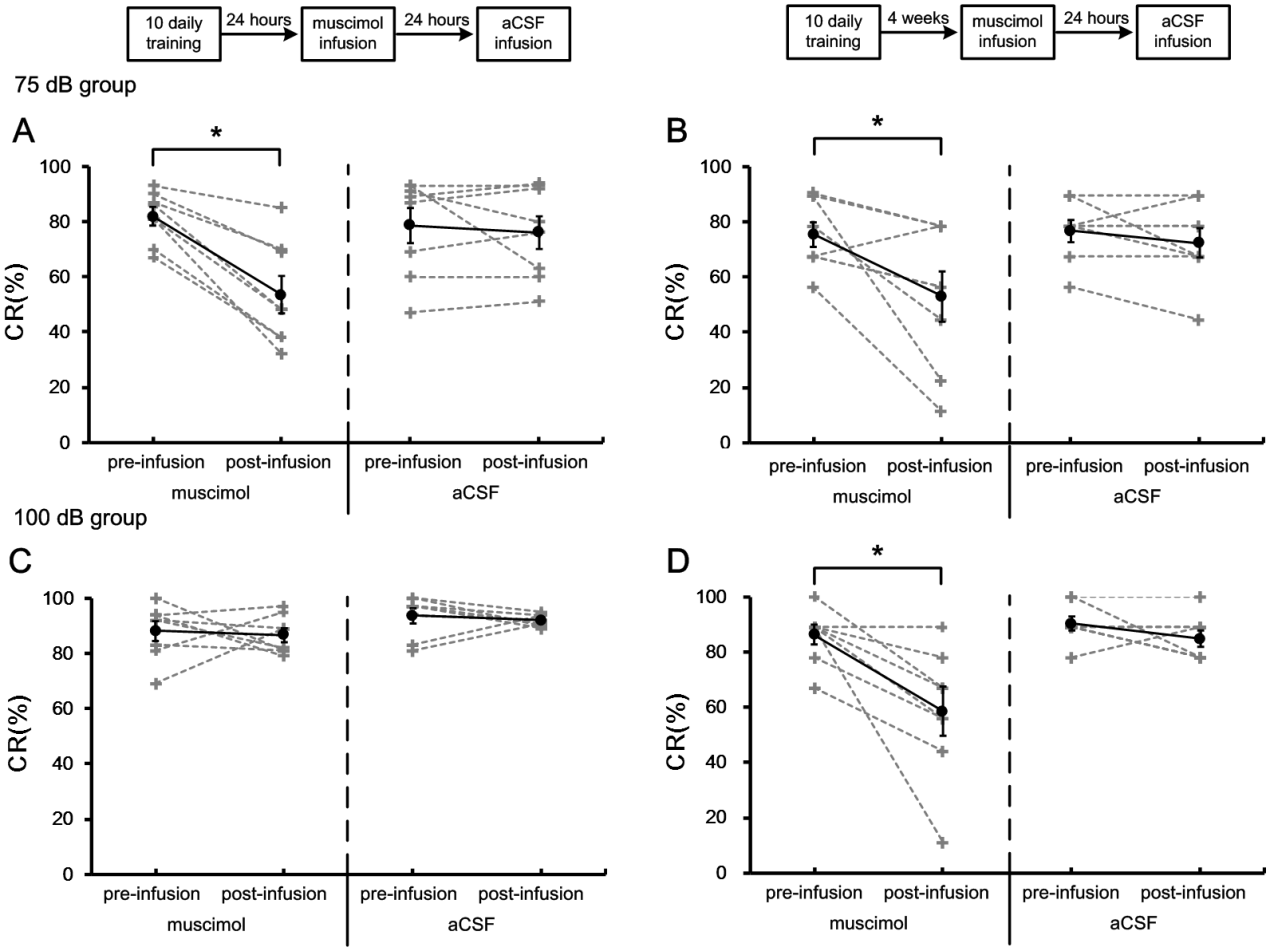
Effects of inactivating cACC on the CR% of 75 dB and 100 dB groups in recent and remote retrieval of tEBC. (A–B) The percentage of CRs of 75 dB group in recent (A) and remote (B) retrieval of tEBC. (C–D) The percentage of CRs of 100 dB group in recent (C) and remote (D) retrieval of tEBC. The data of individuals were indicated by gray dash lines, and the mean values were indicated by black solid lines. Error bars represent the SEM. Muscimol infusion had significant effect on the CR% of 75 dB group in both recent and remote retrieval of tEBC [Paired-Sample T test; recent: p <0.001; remote: p = 0.036]. In 100 dB group, muscimol infusion had significant effect on the CR% in remote but not recent retrieval of tEBC [Paired-Sample T test; recent: p = 0.76; remote: p = 0.011]. *p < 0.05, Paired-Sample T test.

Fig 4 shows the CR% of the 75 dB and 100 dB groups in the retrieval of tEBC. Paired-Sample T test revealed that infusion of muscimol into the cACC significantly decreased the CR% in both recent [muscimol: pre = 82 ± 3.3%, post = 53.5 ± 6.7%, t = 6.235, df = 7, p <0.001; aCSF: pre = 78.8 ± 6.2%, post = 76 ± 5.9%, t = 0.62, df = 7, p = 0.556] and remote [muscimol: pre = 75.1 ± 4.6%, post = 52.8 ± 9.1%, t = 2.59, df = 7, p = 0.036; aCSF: pre = 76.4 ± 3.9%, post = 72.2 ± 5.1%, t = 1.16, df = 7, p = 0.285] retrieval of tEBC with 75 dB tone CS. Infusing muscimol into the cACC significantly decreased the CR% in remote [muscimol: pre = 86.1 ± 3.5%, post = 58.3 ± 8.3%, t = 3.415, df = 7, p = 0.011; aCSF: pre = 90.3 ± 2.5%, post = 84.7 ± 2.9%, t = 1.528, df = 7, p = 0.17] but not recent [muscimol: pre = 88.2 ± 3.5%, post = 86.7 ± 2.4%, t = 0.318, df = 7, p = 0.76; aCSF: pre = 93.8 ± 2.7%, post = 91.9 ± 0.8%, t = 0.678, df = 7, p = 0.519] retrieval of tEBC with 100 dB tone CS.

**Fig 5:**
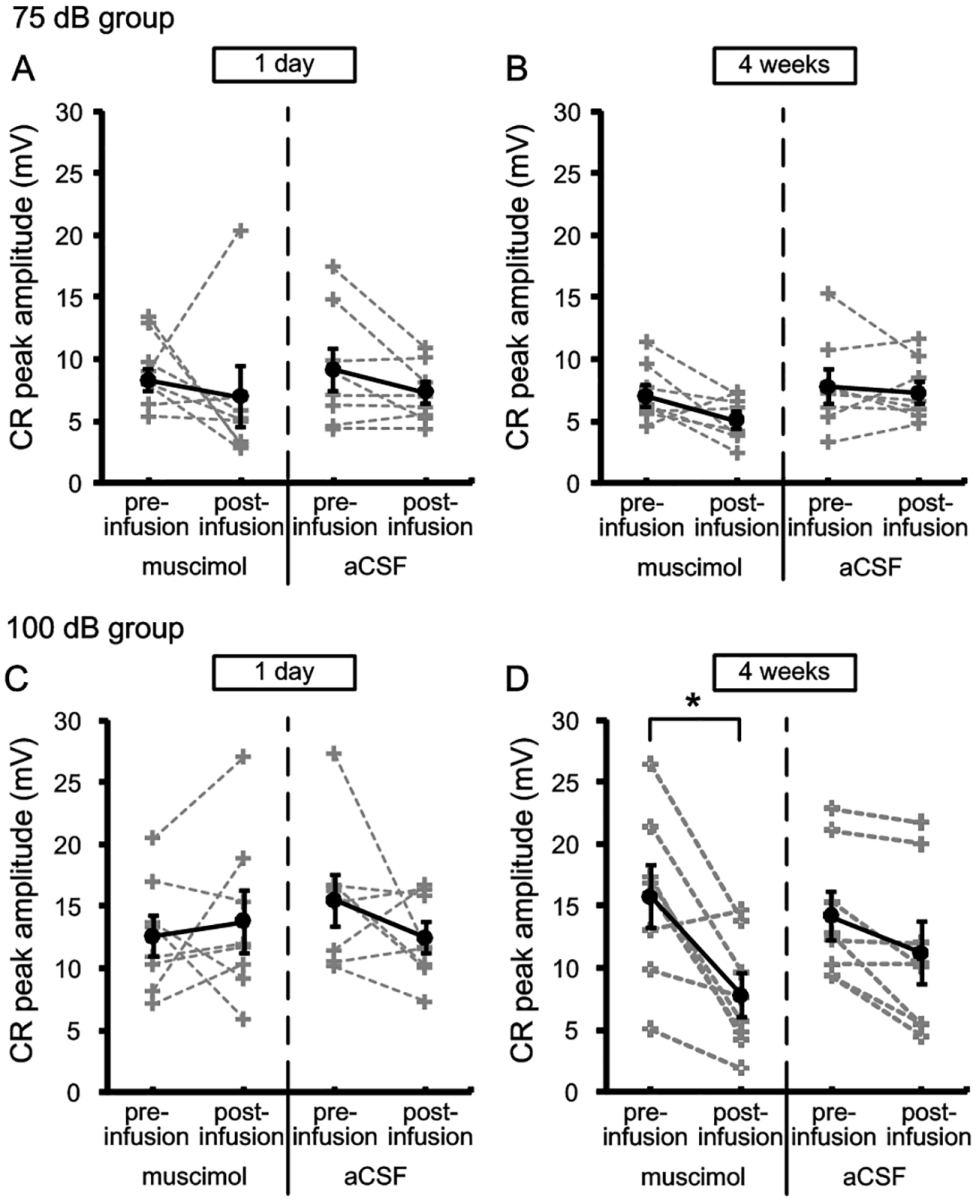
Effects of inactivating cACC on the CR peak amplitude of 75 dB and 100 dB groups in recent and remote retrieval of tEBC. (A–B) The CR peak amplitudes of 75 dB group in recent (A) and remote (B) retrieval of tEBC. (C–D) The CR peak amplitudes of 100 dB group in recent (C) and remote (D) retrieval of tEBC. The data of individuals were indicated by gray dash lines, and the mean values were indicated by black solid lines. Error bars represent the SEM. Muscimol infusion had no significant effect on the CR peak amplitude of 75 dB group in both recent and remote retrieval of tEBC [Paired-Sample T test; recent: p = 0.652; remote: p = 0.078]. In 100 dB group, muscimol infusion had significant effect on the CR peak amplitude in remote but not recent retrieval of tEBC [Paired-Sample T test; recent: p = 0.587; remote: p = 0.014]. *p < 0.05, Paired-Sample T test.

CR peak amplitude and CR peak latency of two groups were analyzed to investigate the effects of the cACC inactivation on the pattern of CR in recent and remote retrieval of tEBC. Fig 5 shows the CR peak amplitude of the 75 dB and 100 dB groups in the retrieval of tEBC. Paired-Sample T test revealed that there were no significant differences in the CR peak amplitude of 75 dB group between the pre-infusion and post-infusion sessions in both recent [muscimol: pre = 8.3 ± 0.9, post = 7 ± 2.3, t = 0.471, df = 7, p = 0.652; aCSF: pre = 9.2 ± 1.6, post = 7.3 ± 0.8, t = 1.79, df = 7, p = 0.117] and remote [muscimol: pre = 7 ± 0.8, post = 5.1 ± 0.6, t = 2.067, df = 7, p = 0.078; aCSF: pre = 7.8 ± 1.3, post = 7.3 ± 0.9, t = 0.573, df = 7, p = 0.585] retrieval of tEBC. In 100 dB group, the CR peak amplitude was significantly decreased after muscimol infusion in remote [muscimol: pre = 19.5 ± 3.3, post = 10.3 ± 3, t = 3.231, df = 7, p = 0.014; aCSF: pre = 14.2 ± 1.9, post = 11.2 ± 2.3, t = 3.076, df = 7, p = 0.18] but not recent retrieval of tEBC [muscimol: pre = 12.6 ± 1.6, post = 13.8 ± 2.3, t = -0.57, df = 7, p = 0.587; aCSF: pre = 15.5 ± 2, post = 12.4 ± 1.2, t = 1.35, df = 7, p = 0.219].

**Fig 6:**
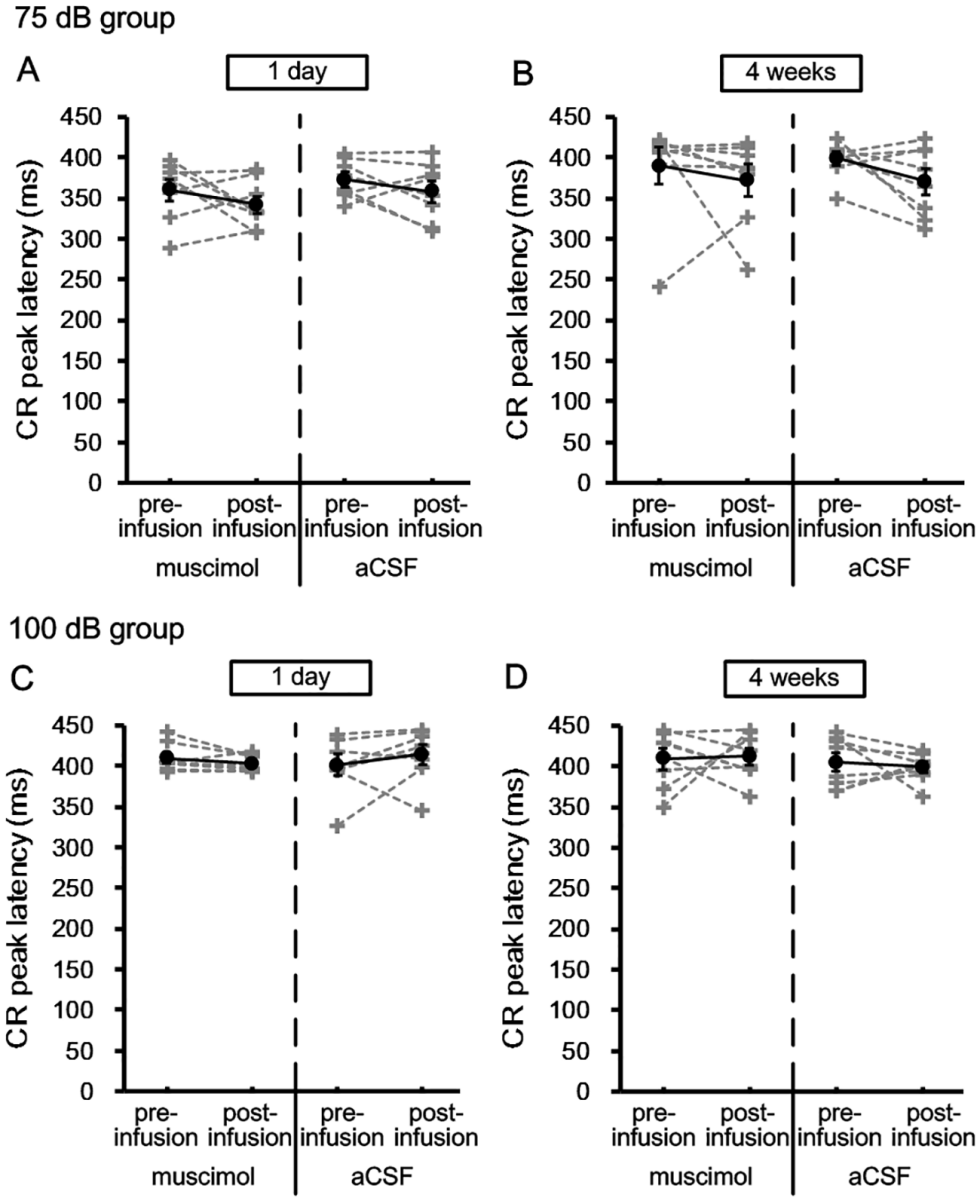
Effects of inactivating cACC on the CR peak latency of 75 dB and 100 dB groups in recent and remote retrieval of tEBC. (A–B) The CR peak latencies of 75 dB group in recent (A) and remote (B) retrieval of tEBC. (C–D) The CR peak latencies of 100 dB group in recent (C) and remote (D) retrieval of tEBC. The data of individuals were indicated by gray dash lines, and the mean values were indicated by black solid lines. Error bars represent the SEM. Muscimol infusion had no significant effect on the CR peak latency of 75 dB and 100 dB group in both recent and remote retrieval of tEBC [Paired-Sample T test; 75 dB group; recent: p = 0.256; remote: p = 0.478; 100 dB group; recent: p = 0.189; remote: p = 0.87]. *p < 0.05, Paired-Sample T test.

Fig 6 shows the CR peak latency of the 75 dB and 100 dB groups in the retrieval of tEBC. Paired-Sample T test revealed that there were no significant differences in the CR peak latency of two group between the pre-infusion and post-infusion sessions in both recent [75 dB group; muscimol: pre = 359.9 ± 12.8, post = 341.8 ± 10.3, t = 1.236, df = 7, p = 0.256; aCSF: pre = 372.8 ± 8.4, post = 358.6 ± 12.5, t = 1.233, df = 7, p = 0.257; 100 dB group; muscimol: pre = 409.7 ± 6, post = 403.3 ± 3.5, t = 1.456, df = 7, p = 0.189; aCSF: pre = 401.1 ± 12.1, post = 414.4 ± 11.5, t = -1.07, df = 7, p = 0.32] and remote [75 dB group; muscimol: pre = 389.8 ± 21.4, post = 371.8 ± 18.5, t = 0.75, df = 7, p = 0.478; aCSF: pre = 398.8 ± 7.8, post = 370.4 ± 15.1, t = 1.861, df = 7, p = 0.105; 100 dB group; muscimol: pre = 409 ± 12, post = 412 ± 10, t = -1.7, df = 7, p = 0.87; aCSF: pre = 404.7 ± 10.9, post = 399 ± 6.2, t = 0.467, df = 7, p = 0.655] retrieval of tEBC.

### Effects of cACC inactivation on the SR

**Fig 7:**
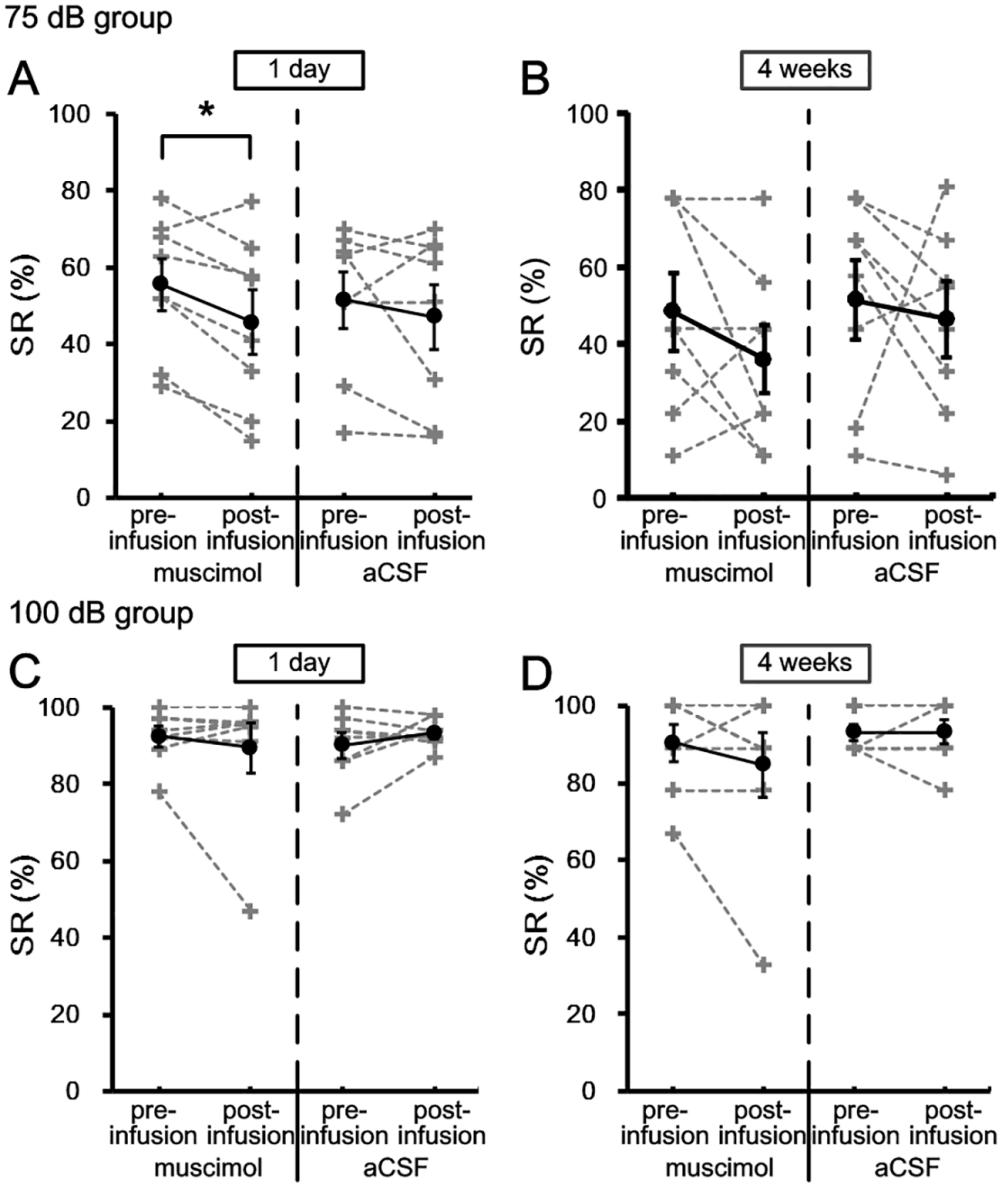
Effects of inactivating cACC on the SR% of 75 dB and 100 dB groups in recent and remote retrieval of tEBC. (A–B) The percentage of SRs of 75 dB group in recent (A) and remote (B) retrieval of tEBC. (C–D) The percentage of SRs of 100 dB group in recent (C) and remote (D) retrieval of tEBC. The data of individuals were indicated by gray dash lines, and the mean values were indicated by black solid lines. Error bars represent the SEM. Muscimol infusion had significant effect on the SR% of 75 dB group in recent but not remote retrieval of tEBC [Paired-Sample T test; recent: p = 0.011; remote: p = 0.208]. Muscimol infusion had no significant effect on the SR% of 100 dB group in both recent and remote retrieval of tEBC [Paired-Sample T test; recent: p = 0.509; remote: p = 0.275]. *p < 0.05, Paired-Sample T test.

Fig 7 shows the SR% of the 75 dB and 100 dB groups in the retrieval of tEBC. Paired-Sample T test revealed that the SR% of 75 dB group was significantly impaired when cACC was inactivated 1 day [muscimol: pre = 55.5 ± 6.3%, post = 45.8 ± 7.8%, t = 3.419, df = 7, p = 0.011; aCSF: pre = 51.5 ± 6.8%, post = 47.1 ± 8%, t = 0.87, df = 7, p = 0.413] but not 4 weeks [muscimol: pre = 48.5 ± 9.4%, post = 36 ± 8.4%, t = 1.387, df = 7, p = 0.208; aCSF: pre = 51.4 ± 9.7%, post = 46.5 ± 9.3%, t = 0.376, df = 7, p = 0.718] after the daily conditioning training. In 100 dB group, cACC inactivation at 1 day [muscimol: pre = 92.4 ± 2.4%, post = 89.5 ± 6.1%, t = 0.696, df = 7, p = 0.509; aCSF: pre = 90.1 ± 3.1%, post = 93.3 ± 1.3%, t = -1.198, df = 7, p = 0.27] or 4 weeks [muscimol: pre = 90.4 ± 4.4%, post = 84.8 ± 7.9%, t = 1.183, df = 7, p = 0.275; aCSF: pre = 93.1 ± 2%, post = 93.1 ± 2.9%, t = 0, df = 7, p = 1] after the daily conditioning has no significant effect on SR%.

## Discussion

In the present study, we investigated the time-limited involvement of the cACC in tEBC mnemonic processes with a 75 dB or 100 dB tone CS. We found that cACC inactivation at 1 day after training impaired the recent retrieval of tEBC with the 75 dB tone CS, but not that of tEBC with the 100 dB tone CS, and that when the cACC was inactivated at 4 weeks after training, the remote retrieval of tEBC in both the 75 dB and 100 dB groups was impaired. These data suggest that the cACC is conditionally involved in recent retrieval of tEBC, depending on the intensity of the CS, but the cACC is persistently required for remote retrieval of tEBC regardless of whether the CS intensity is high or low. In addition, it is notable that in our study cACC inactivation resulted in significant but incomplete abolition of CRs in tEBC, suggesting that the role of the cACC is to modulate but not to mediate tEBC retrieval.

We also noted that compared with animals in the 75 dB group, animals in the 100 dB group show faster CR acquisition, a higher percentage of CRs and higher CR amplitudes, as well as more accurately timed CR peak latencies, indicating better performance in tEBC. This result is consistent with early reports that the acquisition of EBC varies as a direct function of the tone CS intensity [22]. EBC is a typical form of cerebellar learning that requires establishment of a temporal association between the mossy fiber inputs activated by the tone CS and the climbing fiber inputs activated by the reinforcing US [11, 23]. Better learning, demonstrated by a higher percentage of CRs and higher CR amplitudes, as well as more accurate CR peak latencies, in the 100 dB group may be because the 100 dB tone CS may provide greater mossy fiber activation by direct or indirect effects than the 75 dB tone CS does. In addition, the SR, as an indicator of emotional arousal, was much more prominent in the 100 dB group than in the 75 dB group. Differences in emotional arousal level, which tend to induce different levels of responsiveness [24], may contribute to the differences in CR peak amplitudes and CR peak latencies between the two groups.

Many studies have confirmed the time-limited involvement of the mPFC in mnemonic processes [25–27]. It is speculated that as the CS-US association is consolidated over time, brain structures mediating the CR appear to reorganize such that the hippocampus becomes less important and the PFC becomes more important [28–30]. Frankland reported that the ACC of the mPFC plays a critical role in remote but not recent spatial memory and contextual fear memory in mice [31, 32]. In addition, Fanselow [33] reported that both recent and remote contextual fear memories as well as remote trace fear memories were disrupted by mPFC (PL) lesions in rats. In the present study, the inactivated regions were localized in the cACC. We confirmed the persistent involvement of the cACC in remote memory of tEBC and the conditional involvement of the cACC in recent memory of tEBC. In contrast to our results, Oswald reported that the greatest tEBC deficits were found when the ACC was lesioned immediately and the PL was lesioned 1 week following learning [12]. Differences in the actual mPFC subregions being inactivated or in the time points when inactivation was performed in various studies may alter the relative involvement of the mPFC in memory.

Takehara et al. reported that mPFC lesions in rats appear to produce increasing performance deficits in tEBC as the time after tEBC acquisition increases [27]. In that experiment, rats received aspiration lesions of the mPFC (ACC and PL) at 1 day, 2 weeks, or 4 weeks after tEBC training. The greatest performance deficits were observed when lesions were performed at 4 weeks after training, while the least performance deficits were observed with lesions at 1 day post-training. However, Simon reported that time-dependent effects of the mPFC (ACC and PL) on performance of tEBC were not found in rabbits when mPFC lesions were made at 1 day, 1 week, 2 weeks, or 1 month after training [34]. It is noteworthy that, ignoring the distinction between different animal species, the experimental parameters (e.g., CS and US intensities) used in the two studies were different, which may be an explanation for the above-mentioned inconsistency. In the present study, the time-dependent role of the mPFC (cACC) in tEBC retrieval was observed in condition of different CS intensity. Our results therefore support this explanation for the inconsistencies between the previous studies in their findings regarding the time-dependent involvement of the mPFC in tEBC.

Similar to our study, Oswald and colleagues reported that involvement of the mPFC in modulation of tEBC depends on the intensity of the US [13, 14]. Given that a high-intensity US or CS can serve as a strong aversive stimulus that will induce high levels of emotional arousal in animals, it is tempting to postulate that emotional arousal is an important factor affecting the mPFC’s modulation of tEBC. Although emotional arousal is well known to modulate memory, the underlying mechanism is unclear. The amygdala has been cited as an essential part of the neural circuitry for processing emotion, and emotional arousal seems to affect memory by inducing amygdala-related modulation of other brain regions, including the mPFC, hippocampus, and striatum. [35–38]. For example, the mPFC-amygdala circuit plays an important role in emotional memories [35, 39], and the amygdala may provide an additional, parallel conditioned stimulus-associated input to the cerebellum [40]. These results emphasize the important effects of emotional arousal on motor learning and memory.

It is notable that as an unconditioned response, the SR can also be affected by mPFC inactivation immediately after tEBC acquisition when using a 75 dB tone as a CS; however, when using a 100 dB tone as the CS, mPFC inactivation does not affect the SR. There is a remarkable distinction between the effects of the mPFC on the SR, which is unconditioned and stable, and on the CR, which is conditionally acquired and fragile. In addition, it should be noted that mPFC inactivation may reduce the efficacy of attention [41, 42], in turn impairing tEBC. The impaired retrieval of tEBC with the 75 dB tone CS immediately after tEBC acquisition may be partly due to reduced selective attention to the CS.

In summary, the present study demonstrates that the necessity of the cACC of the mPFC for tEBC expression immediately after tEBC acquisition depends on the intensity of the CS, while cACC necessity for the long-term expression of tEBC does not depend on CS intensity. Together with previous studies, the cACC seems to play an attentional role in recent retrieval of tEBC, and the cACC is important for the retrieval of tEBC when emotional arousal is low but not when emotional arousal is high, because some additional brain structures such as hippocampus or amygdala may be activated to support this task when emotional arousal is high. However, in remote retrieval of tEBC, the cACC may play an important role in memory retrieval or memory storage, thus it is critical for the retrieval of tEBC in both arousal conditions.

Our results will contribute to the understanding of mnemonic processes of EBC and underscore the importance of emotional arousal effects on the memory. Further studies are required to explore the mechanisms regarding how arousal affects simple associative motor learning and memory.

## Abbreviations

ACC: anterior cingulate cortex
cACC: caudal anterior cingulate cortex
CR: conditioned response
CS: conditioned stimulus
dEBC: delay eyeblink conditioning
EBC: Eyeblink conditioning
mPFC: medial prefrontal cortex
PL: prelimbic cortex
SR: startle response
tEBC: trace eyeblink conditioning
UR: unconditioned response
US: unconditioned stimulus
